# Systemic biomarkers of inflammation and haemostasis in patients with chronic necrotizing pulmonary aspergillosis

**DOI:** 10.1186/1471-2334-12-144

**Published:** 2012-06-25

**Authors:** Ernst Kristian Rødland, Thor Ueland, Stine Bjørnsen, Ellen Lund Sagen, Christen Peder Dahl, Anne Naalsund, Tom Eirik Mollnes, Frank R Brosstad, Fredrik Müller, Pål Aukrust, Stig S Frøland

**Affiliations:** 1 Research Institute for Internal Medicine, University of Oslo, Oslo, Norway; 2 Section of Clinical Immunology and Infectious Diseases, University of Oslo, Oslo, Norway; 3 Department of Endocrinology, University of Oslo, Oslo, Norway; 4 Department of Respiratory Medicine, University of Oslo, Oslo, Norway; 5 Institute of immunology, University of Oslo, Oslo, Norway; 6 Department of Microbiology, Rikshospitalet, Oslo University Hospital, University of Oslo, Oslo, Norway; 7 Faculty of Medicine, University of Oslo, Oslo, Norway; 8 Research Institute for Internal Medicine, Rikshospitalet, Oslo University Hospital, N-0027, Oslo, Norway

**Keywords:** CNPA, Inflammation, Haemostasis, Biomarkers

## Abstract

**Background:**

The purpose of this study was to investigate mediators of inflammation and haemostasis in patients with chronic necrotizing pulmonary aspergillosis (CNPA), a locally, destructive process of the lung due to invasion by *Aspergillus* species.

**Methods:**

Measurements of selected biomarkers in 10 patients with CNPA and 19 healthy, matched controls were performed with enzyme-linked immunosorbent assay (ELISA) and multiplex methodology. The gene expressions of relevant biomarkers were analyzed with real-time quantitative RT-PCR.

**Results:**

Increased concentrations of circulating mediators of inflammation interleukin (IL)-6, IL-8, RANTES, TNF-α, ICAM-1 and mediators involved in endothelial activation and thrombosis (vWF, TF and PAI-1) were observed in patients with CNPA. The concentration of the anti-inflammatory cytokine IL-10 was increased both in plasma and in PBMC in the patient population. The gene expression of CD40L was decreased in PBMC from the patient group, accompanied by decreased concentrations of soluble (s) CD40L in the circulation.

**Conclusions:**

The proinflammatory response against *Aspergillus* may be counteracted by reduced CD40L and sCD40L, as well as increased IL-10, which may compromise the immune response against *Aspergillus* in patients with CNPA.

## Background

The ubiquitous mould *Aspergillus fumigatus* causes a variety of clinical syndromes ranging from allergic disease in immunocompetent individuals to invasive pulmonary aspergillosis in severely immunocompromised patients [1,2]. Chronic necrotizing pulmonary aspergillosis (CNPA) is a locally destructive process of the lung due to semi invasive infection by *Aspergillus* species often accompanied by development of a fungus ball (aspergilloma). CNPA which is a relatively uncommon form of *Aspergillus* infection, typically occurs as a complication of preexisting chronic lung diseases such as emphysema, sarcoidosis, tuberculosis sequela and other fibrotic lung diseases as well as in mildly immunocompromised patients e.g. diabetes mellitus, chronic liver disease and alcoholism [3,4]. The most common symptoms are chronic, productive cough with or without hemoptysis and pleuritic chest pain, but constitutional symptoms (i.e. weight loss, fever and lack of energy) are also seen [3-5]. Although a few reports suggest a dysregulated cytokine response and involvement of complement activation in CNPA [6], little is known about the immunopathogenic mechanisms of this disorder. Moreover, whereas thrombosis and vascular invasion are histopathological hallmarks of invasive aspergillosis in severely immunocompromised patients [7,8], the role of platelet and endothelial cell activation as well as the levels of prothrombotic mediators in CNPA are largely unknown [4,6]. To further elucidate these issues, we measured selected biomarkers involved in inflammation and thrombosis in 10 patients with CNPA and in 19 sex- and age-matched healthy individuals.

## Materials and methods

### Patients and controls

Between January 2002 and January 2008, 10 patients referred to the Department of Respiratory Medicine, Rikshospitalet, Oslo University Hospital, where a diagnosis of CNPA were established, were included in the study. The study population consisted of two women and eight men, ranging from 52 to 78 years of age (median 63 years) (Table 1).

**Table 1:** Characteristics of the patients with chronic necrotizing pulmonary aspergillosis

**Patient**	**Sex**	**Age**	**Disease**	**All antifungal medication**	**On antifungals when tested**	**Biopsy or BAL**^**1**^	**Serology**^**2**^
#1	Female	61	Sarcoidosis	ITR, VOR	No	Pos.	Neg.
#2	Male	52	AML	ITR, VOR	No	Pos.	Pos.
#3	Male	53	Sarcoidosis	ITR, VOR	No	Pos.	Pos.
#4	Female	63	Tbc	ITR, VOR	Yes	Neg.	Pos.
#5	Male	71	ND	VOR	Yes	Neg.	Pos.
#6	Male	78	ND	None	No	Neg.	Pos.
#7	Male	78	RA	None	No	Pos.	Neg.
#8	Male	63	ND	VOR	No	Pos.	Pos.
#9	Male	57	Emphysema	FLU, AMB, VOR, CAS	Yes	Pos.	Pos.
#10	Male	65	Sarcoidosis	CAS, VOR	No	Pos.	Neg.

AML, treated for acute myelogenic leukemia ten years ago; Tbc, previous pulmonary tuberculosis; ND, no primary pulmonary disorders or pre-disposing immunodeficiency were detected; RA, rheumatoid arthritis; ITR, itraconazole; VOR, voriconazole; FLU, fluconazole; AMB, amphotericin B; CAS, caspofungin.

^1^ Culture or through direct microscopy.

^2^ At time of sampling.

The diagnosis of CNPA was established using clinical examination, radiological manifestations, and microbiological and serological results [9]. Four patients had a history of CNPA for more than 2 years at the time of sampling. All patients had apical, pulmonary changes such as pleural thickening and signs of local parenchymal destruction on radiological examination and aspergillomas were present in all cases. Five of the patients had radiological evidence of fibrosis. Five patients also used a low dose (5–10 mg) prednisolon on a daily basis, and three out of ten were on the antifungal drug voriconazole at the time of sampling. As controls we included blood samples from 19 sex- and age-matched apparently healthy (based on disease history and routine blood tests including C-reactive protein) individuals [ranging from 50 to 73 years (median 60 years); 11 men and 8 women] that were recruited from the same part of Norway as the patients. Except for co-morbidities, there were no demographic differences between the patients and controls. Informed consent was obtained from patients and controls, and the study was approved by Regional Committee for Medical and Research Ethics, South-East, Norway.

### Blood sampling and cell isolation

Platelet-poor plasma and serum were collected and stored as previously described [10]. Peripheral blood mononuclear cells (PBMC) were obtained from heparinized blood by Isopaque-Ficoll (Lymphoprep; Axis Shield, Oslo, Norway) gradient centrifugation and stored in liquid nitrogen until further analyses.

### Measurements of cytokines and markers of platelet and endothelial activation

Plasma levels of interleukin (IL)-6, IL-8/CXCL8, IL-10, monocyte chemoattractant protein (MCP)-1/CCL2, *r*egulated on *a*ctivation, *n*ormal *T* cell *e*xpressed and *s*ecreted RANTES/CCL5 and tumor necrosis factor (TNF)-α were analyzed by Multiplex technology using a customized Bio-Plex Pro Human 8-plex express assay (Bio-Rad Laboratories, Hercules, CA). Serum concentrations of CX3CL1 (fractalkine), intracellular adhesion molecule (ICAM)-1, vascular cell adhesion molecule (VCAM)-1, E-selectin, and plasma concentrations of plasminogen activator inhibitor (PAI)-1 were analyzed by enzyme immunoassays (EIAs) provided from R&D Systems (Minneapolis, MN). Plasma concentrations of tissue factor (TF) and thrombomodulin were analyzed by EIAs (American Diagnostica, Stamford, CT). Plasma concentrations of von Willebrand factor (vWF) and serum concentrations of soluble CD40 ligand (sCD40L) were measured by EIAs obtained from Dako (Glostrup, Denmark) and Bender MedSystems (Vienna, Austria), respectively.

### Complement studies

The concentration of mannose-binding lectin (MBL) was measured by a double antibody enzyme-linked immunosorbent assay (ELISA) as previously described [11]. The determination of functional activity of the classical, lectin and alternative complement pathways of human serum complement, using deposition of the terminal C5b-9 complex in microtiter-wells as read-out (Wielisa®) instead of the traditional red cell hemolytic assays, was performed as previously described [12].

### Real-time quantitative RT-PCR

Total RNA was extracted from PBMC isolated from the CNPA patients and 10 healthy controls using RNeasy columns (Qiagen), subjected to DNase I treatment, and stored in RNA storage solution (Ambion, Austin, TX) at −80 °C. Due to technical difficulties, RNA from one of the patients was not obtainable. Primers for IL-6, IL-8, IL-10, TNF-α, RANTES, CD40, CD40L, ICAM-1, and PAI-1 were designed using the Primer Express software, version 2.0 (Applied Biosystems, Foster City, CA). Primer sequences can be provided by request. Quantification of mRNA was performed using the ABI Prism 7500 (Applied Biosystems). Gene expression of the housekeeping gene β-actin (Applied Biosystems) was used for normalization.

### Statistical analysis

Differences in circulating biomarkers were evaluated by linear regression with biomarker as dependent variable and group (i.e. patients vs. controls) and use of voriconazole as independent variables in a forced model. Differences in mRNA levels of the different markers were analyzed with the Mann–Whitney *U* test. All p-values are two-sided and p < 0.05 was considered significant. Chronic necrotizing aspergillosis is a relatively rare condition, and the present study had an explorative design looking for rather large differences in a relative small population without performing any power calculation.

## Results

### Markers of inflammation and endothelial activation

Several significant differences were observed between CNPA patients (n = 10) and healthy controls (n = 19) with respect to inflammatory and anti-inflammatory mediators as well as markers of endothelial activation.

**Figure 1:**
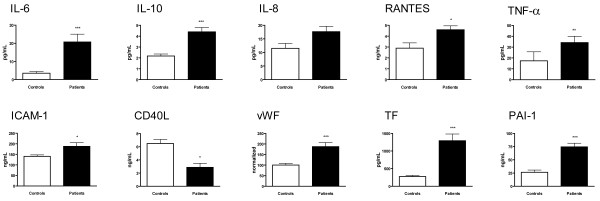
**Plasma and serum levels of IL-6, IL-10, IL-8, RANTES, TNF-α, ICAM-1, soluble CD40L, vWF, tissue factor (TF), and PAI-1 in patients with CNPA (n = 10, black columns) and healthy controls (n = 19, white columns).** Data are mean ± SEM. P- values are adjusted for the use of voriconazole. *p < 0.05, **p < 0.01 and ***p < 0.001 versus controls.

**Figure 2:**
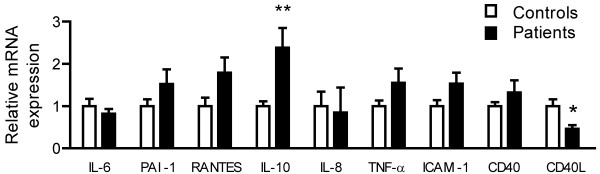
**mRNA expression of IL-6, PAI-1, RANTES, IL-10, IL-8, TNF-a, ICAM-1, CD40, and CD40L in 9 patients with CNPA and 10 healthy controls as assessed by real-time quantitative RT-PCR.** Data are mean ± SEM in relation to the control gene β-actin and healthy controls are normalized equal to 1. *p < 0.05 and **p < 0.01 versus controls.

First, CNPA patients showed increased concentrations of several circulating inflammatory markers including cytokines [IL-6 (p < 0.001) and TNF-α (p < 0.01)], chemokines [IL-8 (not significant, p = 0.057) and RANTES (p < 0.05)], and the adhesion molecule ICAM-1 (p < 0.05) (Figure 1). Four patients with a history of more than 2 years had reduced levels of IL-8 compared with the other patient population, but the difference did not reach statistical significance (p = 0.056). mRNA levels of RANTES were also increased in the CNPA group, although the difference did not reach statistical significance (p = 0.05, Figure 2). As for MCP-1 and CX3CL1, there were no differences between the patients and controls (data not shown).

Second, CNPA patients also had markedly raised levels of the prototypical anti-inflammatory cytokine IL-10 (p < 0.001) (Figure 1). The significant increase in plasma levels of IL-10 was accompanied by an increased mRNA level of this cytokine in PBMC (Figure 2).

Finally, while there was a marked increase in levels of circulating vWF (p < 0.001) in patients with CNPA compared with healthy controls (Figure 1), other parameters of endothelial cell activation (i.e., VCAM-1 and E-selectin) were not different between the two groups (data not shown).

### Markers of haemostasis

CNPA patients had significantly raised plasma levels of the pro-thrombotic mediators TF (p < 0.001) and PAI-1 (p < 0.001) (Figure 1). In contrast, the anti-thrombotic mediator thrombomodulin showed no difference between CNPA patients and controls (data not shown).

### CD40/CD40L

Serum levels of sCD40L were significantly decreased (p < 0.05) in the CNPA group as compared with healthy controls (Figure 1). Patients with CNPA also had significant decreased mRNA levels of CD40L in PBMC (Figure 2).

### Complement parameters in plasma

All patients and controls had normal activity in the classical and alternative complement pathways with no differences between the two groups (data not shown). Two patients and one healthy control had a total defect in the lectin pathway with MBL levels <50 ng/mL, but there were no significant differences in MBL levels between the groups (data not shown). There were no individuals in our material who were partly deficient in MBL (50–400 ng/mL).

### Effect of therapy and fibrosis on biomarker levels

All selected biomarkers were evaluated to assess if the use of voriconazole (n = 3), prednisolon (n = 5), evidence of fibrosis (n = 5) or duration of disease ≤ 2 years (n = 6) vs. > 2 years (n = 4) influenced the results. No such effects were observed (data not shown). However, when including voriconazol in the analysis as an independent variable in a forced model, the difference between patients and controls for IL-8 did not reach statistical significance (p = 0.057).

## Discussion

CNPA is characterized by persistent inflammation caused by an unresolved infection possibly reflecting an altered antifungal immune response. Several names have been proposed for different entities of this condition [3] as it is thought to represent a spectrum of diseases depending on the degree of invasion of pulmonary parenchyma, formation and expansion of multiple cavities over time and the development of fibrosis in surrounding tissue [13]. The term CNPA is often used to describe all entities in this spectrum of illness.

CD40-CD40L interaction regulates several aspects of cell-mediated immunity, including activation of antigen-presenting cells, CD4^+^ and CD8^+^ T cells, and stimulation of IL-12/IFN-γ production [14,15]. The CD40-CD40L axis has been implicated in anti-fungal defense mechanisms [16-20]. Notably, the patients with CNPA had decreased expression of the gene encoding CD40L in PBMC and decreased levels of sCD40L. sCD40L is primarily derived from platelets [21-23]. Since *A. fumigatus* has been shown to induce platelet activation [24,25], low serum sCD40L in CNPA patients could reflect degranulated platelets secondary to chronic activation. On the other hand, the reduced expression of CD40L in PBMC may suggest a more general impairment in the CD40-CD40L axis in patients with CNPA, possibly contributing to a decreased ability to clear the fungus in these patients as has been demonstrated in other mycoses [16-18,20].

A major observation in the present study was the markedly increased IL-10 levels in the CNPA group as seen both in serum and mRNA expression in PBMC. IL-10 may have suppressive effects on the antifungal activity of mononuclear cells against *A. fumigatus* in vitro [6], but the role of IL-10 in pulmonary *Aspergillus* manifestations is still debated. It is conceivable that high IL-10 levels could contribute to the resolution of the *Aspergillus* induced pulmonary inflammation, but high IL-10 concentrations have also been associated with development of invasive aspergillosis in non-neutropenic immunocompromised patients and with colonization with *A. fumigatus* and allergic bronchopulmonary aspergillosis in patients with cystic fibrosis [26]. It is therefore tempting to hypothesize that the net effect of the high IL-10 levels in CNPA patients is to contribute to the maintenance of persistent *Aspergillus* infection within the pulmonary tissue in these patients, inducing a state of nonresolving inflammation.

We further show that patients with CNPA are characterized by significantly increased serum and plasma levels of several inflammatory markers including inflammatory cytokines (IL-6 and TNF-α), chemokines (IL-8 and RANTES) and the adhesion molecule ICAM-1. The reduced levels, although not significant, of IL-8 in patients with a history of CNPA more than 2 years may reflect a down-regulation of the inflammatory response with sustained infection. Although CNPA is a localized disease with only involvement of pulmonary tissue, several of the patients have constitutional symptoms which may be associated with the signs of chronic inflammation reflected in our findings.

Complement defects, and in particular low levels of MBL, have also been related to the development of CNPA [27]. In the present study we did not find any differences in MBL levels between CNPA patients and controls. However, in our opinion, the present study is too small to make any firm conclusion concerning the potential role of MBL.

*Aspergillus fumigatus* is in general angioinvasive, causing intravascular thrombosis and dissemination of the fungus through the blood stream [7,8]. Thrombosis and vascular events have also been documented in patients with CNPA [28]. Our population of CNPA patients had increased levels of the pro-thrombotic mediators TF and PAI-1, without any changes in the anti-thrombotic mediator thrombomodulin. In addition, the CNPA patients showed signs of enhanced endothelial cell activation as assessed by increased vWF levels. The normal concentration of E-selectin might seem in conflict with this finding, but it has previously been shown that the expression of E-selectin on the endothelium may be differently regulated than other markers of endothelial cell activation [29].

Formulations of amphotericin B are proinflammatory stimulants of innate immune cells and the echinocandins have been shown to enhance the antifungal properties of neutrophiles and macrophages [30]. Azoles are the least active to modulate the host`s antifungal defences [30], but voriconazole has nevertheless been shown to induce production of TNF-α in the human, monocytic cell line THP-1 in vitro [31]. In the present study, three out of ten patients used voriconazole at the time of sampling. However, we did not observe any differences in the levels of measured biomarkers, although limited by low numbers, when comparing patients using voriconazole with those who did not. However, when including voriconazole as an independent variable, the difference between patients and controls did not reach statistical significance for IL-8, suggesting some influence of this medication on the immune response in CNPA.

Our findings suggest a possible dysfunction of the CD40-CD40L axis, thus compromising the immune response against *Aspergillus* in patients with CNPA. Elevated levels of the anti-inflammatory cytokine IL-10 may well contribute to an impaired antifungal response. Both findings may predispose to persistence of the fungal infection and unresolved inflammation. CNPA patients have signs of endothelial activation and a pro-thrombotic phenotype, possibly contributing to the well known histopathology of infections with *A. fumigatus*.

## Competing interests

Potential conflicts of interest: none reported.

Financial support: Research Council of Norway, University of Oslo, Medinnova Foundation, and Helse Sør-Øst.

## Authors’ contributions

EKR is the main author and carried out several of the experiments. TU performed the statistical analysis. SB, ELS and TEM carried out most of the experiments. CPD and AN participated in collecting biological material. FRB, FM, PA and SSF conceived of the study, and participated in its design and drafted the manuscript. All authors read and approved the final manuscript.

## Pre-publication history

The pre-publication history for this paper can be accessed here:

http://www.biomedcentral.com/1471-2334/12/144/prepub
